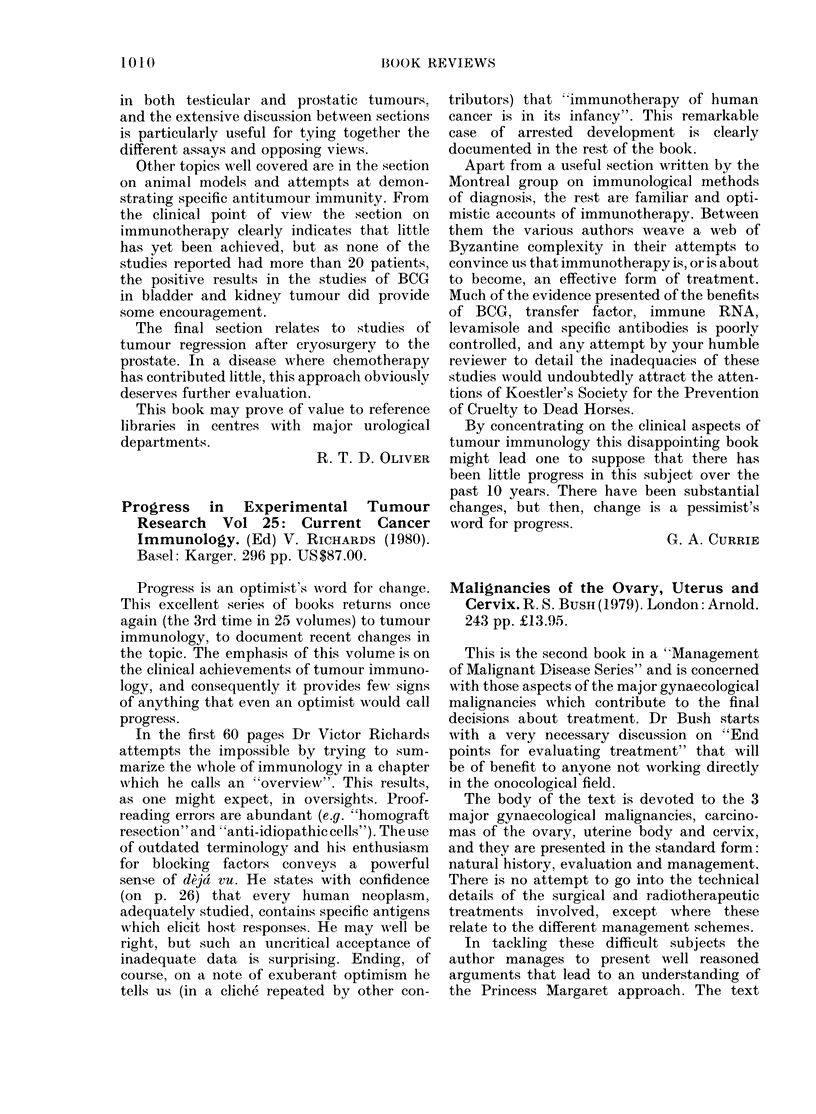# Progress in Experimental Tumour Research Vol 25: Current Cancer Immunology

**Published:** 1980-06

**Authors:** G. A. Currie


**Progress in Experimental Tumour Research Vol 25: Current Cancer Immunology.** (Ed) V. RICHARDS (1980). Basel: Karger. 296 pp. US$87.00.

Progress is an optimist's word for change. This excellent series of books returns once again (the 3rd time in 25 volumes) to tumour immunology, to document recent changes in the topic. The emphasis of this volume is on the clinical achievements of tumour immunology, and consequently it provides few signs of anything that even an optimist would call progress.

In the first 60 pages Dr Victor Richards attempts the impossible by trying to summarize the whole of immunology in a chapter which he calls an "overview". This results, as one might expect, in oversights. Proofreading errors are abundant (*e.g.* "homograft resection" and "anti-idiopathic cells"). The use of outdated terminology and his enthusiasm for blocking factors conveys a powerful sense of *dèjá vu*. He states with confidence (on p. 26) that every human neoplasm, adequately studied, contains specific antigens which elicit host responses. He may well be right, but such an uncritical acceptance of inadequate data is surprising. Ending, of course, on a note of exuberant optimism he tells us (in a cliché repeated by other contributors) that "immunotherapy of human cancer is in its infancy". This remarkable case of arrested development is clearly documented in the rest of the book.

Apart from a useful section written by the Montreal group on immunological methods of diagnosis, the rest are familiar and optimistic accounts of immunotherapy. Between them the various authors weave a web of Byzantine complexity in their attempts to convince us that immunotherapy is, or is about to become, an effective form of treatment. Much of the evidence presented of the benefits of BCG, transfer factor, immune RNA, levamisole and specific antibodies is poorly controlled, and any attempt by your humble reviewer to detail the inadequacies of these studies would undoubtedly attract the attentions of Koestler's Society for the Prevention of Cruelty to Dead Horses.

By concentrating on the clinical aspects of tumour immunology this disappointing book might lead one to suppose that there has been little progress in this subject over the past 10 years. There have been substantial changes, but then, change is a pessimist's word for progress.

G. A. CURRIE